# Supplementary material for: The Societal Value of Vaccines: Expert-Based Conceptual Framework and Methods Using COVID-19 Vaccines as a Case Study
Source: Vaccines (Basel). 2023 Jan 20;11(2):234. doi: 10.3390/vaccines11020234 (PMC9961127; doi:10.3390/vaccines11020234)
Supplement: Supplementary file 1 [file vaccines-11-00234-s001.zip › Supplementary material_S1.pdf]

Supplementary Material S1: Questionnaire for Round 1 of Individual Expert Elicitation

This questionnaire aims to gather initial inputs and perspectives on the topics of the Expert Panel.

Please provide your responses after reviewing the pre-read materials.

Your insights will support preparations for the upcoming panel discussions. The responses will be anonymous and analysed at aggregated level; the results will be used solely for the purpose of this piece of research.

Thank you.

* Required

Demographic questions

1. In which country do you conduct research on? *
   - UK
   - US
   - Other:

2. What is your functional expertise? (Can select multiple options)
   - Health policy
   - Epidemiology
   - Health Technology Assessment
   - Public Health
   - Immunisation
   - Public finance
   - Patient advocacy group
   - Health economics
   - Paediatrics / Child health
   - Other:

3. What type of organisation do you work in or advise?
   - Government Institution
   - Academic Institution e.g., University
   - Patient Association
   - Other:

4. For how many years have you worked in healthcare research or sector? *

5. Have you applied a broader perspective in your prior work? *

NB. This question applies only to experts with previous experience of economic evaluations (e.g., health economists), please select 'Not applicable' otherwise.

- No
- Yes, in prior vaccine evaluations only
- Yes, in vaccine and non-vaccine specific evaluations
- Yes, in non-vaccine specific evaluations only
- Not applicable
- Other

Effects of COVID-19 on broader economic and societal outcomes

Research objective: to identify the key societal outcomes impacted by COVID-19 and value elements

6. Please rank the value elements below according to their relevance or priority for inclusion in economic evaluations of vaccines against COVID- 19 (1 low, 5 high) (1/2) *

Definitions of the elements of value of vaccination below are provided in the pre-read part 1 (slides 19-22).

Please state your opinion independently of the perspective recommended by HTA bodies in your country.

	1	2	3	4	5
A1. Impact on length of life and quality of life of patients	○	○	○	○	○
B1.1 Impact on quality of life of carers	○	○	○	○	○
B1.2 Impact on quality of life of other individuals	○	○	○	○	○
B2. Transmission	○	○	○	○	○
B3. Burden of disease	○	○	○	○	○
B4. Value to other interventions	○	○	○	○	○
B5. AMR Prevention	○	○	○	○	○
B6. Mental health impact	○	○	○	○	○
B7. Health impact of congestion externality	○	○	○	○	○
B8. Health equity	○	○	○	○	○
C1.1. Avoided care cost of infected patients	○	○	○	○	○
C1.2. Avoided care costs related to broad health effects	○	○	○	○	○
C2. Financial sustainability and programmatic synergies	○	○	○	○	○
C3. Public sector budget impact	○	○	○	○	○
D1.1 Impact on patient productivity	○	○	○	○	○
D1.2 Impact on carer productivity	○	○	○	○	○
D1.3 Impact on productivity of other individuals	○	○	○	○	○

	1	2	3	4	5
D2. Impact on costs of non- pharmaceutic al interventions	◯	◯	◯	◯	◯
D3.1 Impact on foregone education of patient	◯	◯	◯	◯	◯
D3.2 Impact on foregone education of other individuals	◯	◯	◯	◯	◯

7. Please rank the value elements below according to their relevance or priority for inclusion in economic evaluations of vaccines against COVID- 19 (1 low, 5 high) (2/2) *

Definitions of the elements of value of vaccination below are provided in the pre-read part 1 (slides 19-22).

Please state your opinion independently of the perspective recommended by HTA bodies in your country.

	1	2	3	4	5
D4. Changes in household behaviour	◯	◯	◯	◯	◯
D5. Macroeconomic effects	◯	◯	◯	◯	◯
D6. Income equity	◯	◯	◯	◯	◯
D7. Scientific spill-over effects	◯	◯	◯	◯	◯
D8. Environmental Effects	◯	◯	◯	◯	◯
E1. Insurance Value	◯	◯	◯	◯	◯
E2. Real option value	◯	◯	◯	◯	◯
E3.1 Value of hope	◯	◯	◯	◯	◯
E3.2 Value of knowing	◯	◯	◯	◯	◯
E3.3 Fear of Diseases	◯	◯	◯	◯	◯

8. What main criteria have you considered for prioritizing the elements of value above? *

9. In your opinion, are there any other elements of value <u>not listed</u> above which should be considered in economic evaluations of vaccines against COVID-19?

If so, please provide the rationale for inclusion and where you would rank them. *

Please state your opinion independently of the perspective recommended by HTA bodies in your country.

10. Please briefly explain if and why some of the elements of value above would <u>NOT</u> be conceptually appropriate to consider *

This question is only applicable where answer to previous questions is 'Yes, some outcomes above would not be appropriate '

11. In your opinion, what is the quality of evidence* supporting the inclusion of outcomes listed below in vaccine assessments? (1/2) *

Please refer to the pre-read materials and rank each element of value from 1 (lowest) to 5 (highest).

*The assessment of a study quality includes contextual, pragmatic and methodological considerations to establish how near the 'truth' its findings are likely to be and if the findings are of relevance in the particular setting or patient group of interest. Quality assessment considers appropriateness of study design to the research objective, risk of bias, statistical issues, generalizability, among others (CRD, University of York, 2009) [1].

	1	2	3	4	5
A1. Impact on length of life and quality of life of patients	◯	◯	◯	◯	◯
B1.1 Impact on quality of life of carers	◯	◯	◯	◯	◯
B1.2 Impact on quality of life of other individuals	◯	◯	◯	◯	◯
B2. Transmission	◯	◯	◯	◯	◯
B3. Burden of disease	◯	◯	◯	◯	◯
B4. Value to other interventions	◯	◯	◯	◯	◯
B5. AMR Prevention	◯	◯	◯	◯	◯
B6. Mental health impact	◯	◯	◯	◯	◯
B7. Health impact of congestion externality	◯	◯	◯	◯	◯
B8. Health equity	◯	◯	◯	◯	◯
C1.1. Avoided care cost of infected patients	◯	◯	◯	◯	◯
C1.2. Avoided care costs related to broad health effects	◯	◯	◯	◯	◯
C2. Financial sustainability and programmatic synergies	◯	◯	◯	◯	◯
C3. Public sector budget impact	◯	◯	◯	◯	◯
D1.1 Impact on patient productivity	◯	◯	◯	◯	◯
D1.2 Impact on carer productivity	◯	◯	◯	◯	◯
D1.3 Impact on productivity of other individuals	◯	◯	◯	◯	◯
D2. Impact on costs of non- pharmaceutic al interventions	◯	◯	◯	◯	◯
D3.1 Impact on foregone education of patient	◯	◯	◯	◯	◯
D3.2 Impact on foregone education of other individuals	◯	◯	◯	◯	◯

12. In your opinion, what is the **quality of evidence*** supporting the inclusion of outcomes listed below in vaccine assessments? (2/2) *

Please refer to the pre-read materials and rank each element of value from 1 (lowest) to 5 (highest).

*The assessment of a study quality includes contextual, pragmatic and methodological considerations to establish how near the 'truth' its findings are likely to be and if the findings are of relevance in the particular setting or patient group of interest. Quality assessment considers appropriateness of study design to the research objective, risk of bias, statistical issues, generalizability, among others (CRD, University of York, 2009).

	1	2	3	4	5
D4. Changes in household behaviour	◯	◯	◯	◯	◯
D5. Macroeconomic effects	◯	◯	◯	◯	◯

	1	2	3	4	5
D6. Income equity	◯	◯	◯	◯	◯
D7. Scientific spill-over effects	◯	◯	◯	◯	◯
D8. Environmental Effects	◯	◯	◯	◯	◯
E1. Insurance Value	◯	◯	◯	◯	◯
E2. Real option value	◯	◯	◯	◯	◯
E3.1 Value of hope	◯	◯	◯	◯	◯
E3.2 Value of knowing	◯	◯	◯	◯	◯
E3.3 Fear of Diseases	◯	◯	◯	◯	◯

13. In your opinion, is the inclusion of the outcomes listed below **likely feasible***? (1/2) *

Please refer to the pre-read materials and rank each element of value from 1 (lowest) to 5 (highest).

*Feasibility defined as: existing methodological approaches would allow the inclusion of societal outcomes in economic models

	1	2	3	4	5
A1. Impact on length of life and quality of life of patients	◯	◯	◯	◯	◯
B1.1 Impact on quality of life of carers	◯	◯	◯	◯	◯
B1.2 Impact on quality of life of other individuals	◯	◯	◯	◯	◯
B2. Transmission	◯	◯	◯	◯	◯
B3. Burden of disease	◯	◯	◯	◯	◯
B4. Value to other interventions	◯	◯	◯	◯	◯
B5. AMR Prevention	◯	◯	◯	◯	◯
B6. Mental health impact	◯	◯	◯	◯	◯
B7. Health impact of congestion externality	◯	◯	◯	◯	◯
B8. Health equity	◯	◯	◯	◯	◯
C1.1. Avoided care cost of infected patients	◯	◯	◯	◯	◯
C1.2. Avoided care costs related to broad health effects	◯	◯	◯	◯	◯
C2. Financial sustainability and programmatic synergies	◯	◯	◯	◯	◯
C3. Public sector budget impact	◯	◯	◯	◯	◯
D1.1 Impact on patient productivity	◯	◯	◯	◯	◯
D1.2 Impact on carer productivity	◯	◯	◯	◯	◯
D1.3 Impact on productivity of other individuals	◯	◯	◯	◯	◯
D2. Impact on costs of non- pharmaceutic al interventions	◯	◯	◯	◯	◯
D3.1 Impact on foregone education of patient	◯	◯	◯	◯	◯
D3.2 Impact on foregone education of other individuals	◯	◯	◯	◯	◯

14. In your opinion, is the inclusion of the outcomes listed below **likely feasible***? (2/2) *

Please refer to the pre-read materials and rank each element of value from 1 (lowest) to 5 (highest).

*Feasibility defined as: existing methodological approaches would allow the inclusion of societal outcomes in economic models

	1	2	3	4	5
D4. Changes in household behaviour	◯	◯	◯	◯	◯
D5. Macroeconomic effects	◯	◯	◯	◯	◯
D6. Income equity	◯	◯	◯	◯	◯
D7. Scientific spill-over effects	◯	◯	◯	◯	◯
D8. Environmental Effects	◯	◯	◯	◯	◯
E1. Insurance Value	◯	◯	◯	◯	◯
E2. Real option value	◯	◯	◯	◯	◯
E3.1 Value of hope	◯	◯	◯	◯	◯
E3.2 Value of knowing	◯	◯	◯	◯	◯
E3.3 Fear of Diseases	◯	◯	◯	◯	◯

15. Which of the outcomes below are currently already routinely included within an assessment by HTA bodies in your country? (1/2)

For those not included, please select option for main rationale for exclusion *

Please state country of relevance in subsequent question.

	Routinely Included in HTA	Not Included in HTA due to lack of robust supporting evidence	Not Included in HTA due to lack of data/difficult to quantify	Not Included in HTA due to lack of ability/capacity to assess	Not Included in HTA due to lack of willingness (not recognised as relevant or appropriate)	Don't know
A1. Impact on length of life and quality of life of patients						
B1.1 Impact on quality of life of carers						
B1.2 Impact on quality of life of other individuals						
B2. Transmission						
B3. Burden of disease						
B4. Value to other interventions						
B5. AMR Prevention						
B6. Mental health impact						
B7. Health impact of congestion externality						
B8. Health equity						
C1.1. Avoided care cost of infected patients						
C1.2. Avoided care costs related to broad health effects						
C2. Financial sustainability and programmatic synergies						
C3. Public sector budget impact						
D1.1 Impact on patient productivity						
D1.2 Impact on carer productivity						
D1.3 Impact on productivity of other individuals						
D2. Impact on costs of non- pharmaceutic al interventions						
D3.1 Impact on foregone education of patient						
D3.2 Impact on foregone education of other individuals						

16. Which of the outcomes below are currently already routinely included within an assessment by HTA bodies in your country? (2/2)

For those not included, please select option for main rationale for exclusion *

Please state country of relevance in subsequent question

	Routinely Included in HTA	Not Included in HTA due to lack of robust supporting evidence	Not Included in HTA due to lack of data/difficult to quantify	Not Included in HTA due to lack of ability/capacity to assess	Not Included in HTA due to lack of willingness (not recognised as relevant or appropriate)	Don't know
D4. Changes in household behaviour	◯	◯	◯	◯	◯	◯
D5. Macroeconomic effects	◯	◯	◯	◯	◯	◯
D6. Income equity	◯	◯	◯	◯	◯	◯
D7. Scientific spill-over effects	◯	◯	◯	◯	◯	◯
D8. Environmental Effects	◯	◯	◯	◯	◯	◯
E1. Insurance Value	◯	◯	◯	◯	◯	◯
E2. Real option value	◯	◯	◯	◯	◯	◯
E3.1 Value of hope	◯	◯	◯	◯	◯	◯
E3.2 Value of knowing	◯	◯	◯	◯	◯	◯
E3.3 Fear of Diseases	◯	◯	◯	◯	◯	◯

17. Please enter country of reference for question 15/16. *

18. Are there any additional barriers to inclusion of the outcomes listed in previous question? If so, what could be potential solutions? *

19. Please share any examples of evidence or good practice in the broad value assessment of vaccines, relating to any of the outcomes above. *

These might be examples from your own country or anywhere else in the world. The examples will be used to help moderate the workshop discussions on overcoming barriers to the inclusion of key outcomes in value assessments of vaccines.

Methods for inclusion of broader value elements

Research objective: to identify methodological approaches to include key societal outcomes impacted by COVID-19 in economic evaluations of vaccines

20. Are the following suitable methods/approaches for inclusion of broader value elements within an assessment of the societal value of vaccination against COVID-19? *

Rank from 1 to 5 based on the relative appropriateness (1 low, 5 high). Methods listed below are described in pre-read part 1 (slides 29 to 31).

Please state your opinion independently of the perspective recommended by HTA bodies in your country.

	1	2	3	4	5	Unsure
B5. Antimicrobial resistance (AMR)- extensions to CEA or CBA	◯	◯	◯	◯	◯	◯
B6. & C1.2. Mental health impact- Approach based on number of additional depression cases	◯	◯	◯	◯	◯	◯
B6. & C1.2. Mental health impact- Approach based on impact of vaccination on months spent in depression	◯	◯	◯	◯	◯	◯
B7. & C1.2 Congestion externality - Opportunity cost	◯	◯	◯	◯	◯	◯
C3. Public finance impact- return on investment (ROI)	◯	◯	◯	◯	◯	◯
C3. Public finance impact- fiscal benefit to cost ratio (fBCR)	◯	◯	◯	◯	◯	◯
D1. Productivity loss - Human capital	◯	◯	◯	◯	◯	◯
D1. Productivity loss - Friction cost	◯	◯	◯	◯	◯	◯
D2. Impact on the cost of non- pharmaceutic al interventions (NPIs)- Approach based on relationship between vaccination and NPI levels	◯	◯	◯	◯	◯	◯
D3. Education loss- Approach based on impact on test scores	◯	◯	◯	◯	◯	◯
D3. Education loss- Microsimulation	◯	◯	◯	◯	◯	◯
D5. Impact on GDP- Approach relies on external estimates for GDP	◯	◯	◯	◯	◯	◯
D5. Impact on GDP- Simple estimate using time series data	◯	◯	◯	◯	◯	◯
D5. Impact on GDP- Macroeconomic modelling	◯	◯	◯	◯	◯	◯

21. Are there any methods not listed above that should be considered? *

Please state any relevant methods, respective value elements and envisaged advantages if applicable

22. If any method listed above would not be suitable or unclear, please explain why *

23. Open feedback on the Survey

Please share your feedback around the usability, design, wording, terminology and clarity of the questions in this questionnaire
