# Supplementary material for: The Societal Value of Vaccines: Expert-Based Conceptual Framework and Methods Using COVID-19 Vaccines as a Case Study
Source: Vaccines (Basel). 2023 Jan 20;11(2):234. doi: 10.3390/vaccines11020234 (PMC9961127; doi:10.3390/vaccines11020234)
Supplement: Supplementary file 1 [file vaccines-11-00234-s001.zip › Supplementary material_S2.pdf]

Supplementary Material S2: COVID-19 Impact Inventory

Table S1: COVID-19 Impact Inventory

Original (Panel 1) CATEGORY	Report CATEGORY	Original (Panel 1) DEFINITION	Report definition	Change
**A. Narrow Health Effects**	**A. Narrow Health Effects**	Impact of vaccines on the health of vaccinated individuals	Impact of vaccines on the health of vaccinated individuals	-
**A1. Impact on length of life and QoL of patients**	**A1. Impact on length of life and QoL of patients**	Impact on life expectancy or life-years saved, and on patients' physical, mental, emotional, and social functioning	Impact on life expectancy or life-years saved, and on patients' physical, mental, emotional, and social functioning, including mortality and QoL impact of potential adverse events related to vaccination. Definition based on Deogaonkar (2012)	Definition expanded to include QoL impact of adverse events related to vaccination
**B. Broad Health Effects**	**B. Broad Health Effects**	Impact of vaccines on the health of the unvaccinated population	Impact of vaccines on the health of the unvaccinated population	-
**B1. Impact on QoL**<br>**B1.1 Impact on QoL of carers**<br>**B1.2 Impact on QoL of other individuals**	**B1. Impact on QoL**<br>**B1.1 Impact on QoL of carers**<br>**B1.2 Impact on QoL of other individuals**	Impact on caregivers' and other individuals' physical, mental, emotional, and social functioning	Impact on caregivers' and other individuals' physical, mental, emotional, and social functioning	-
**B2. Transmission value**	**B2. Transmission value**	Impact on disease transmission patterns and associated morbidity and mortality	Impact on disease transmission patterns and associated morbidity and mortality	-
**B3. Burden of disease**	**B3. Burden of disease**	Impact on overall burden of disease to society, in terms of prevalence and severity, estimated through the total amount of associated morbidity and mortality<br>(Note, this includes A1., B1., and B2.)	Impact on overall burden of disease to society, in terms of prevalence and severity, estimated through the total amount of associated morbidity and mortality<br>(Note, this includes A1., B1., and B2.)	-
**B4. Value to other interventions**	**B4. Value to other interventions**	Impact on the cost effectiveness of other non-vaccine interventions (also referred to as enablement value)	Impact on the cost effectiveness of other non-vaccine interventions (also referred to as enablement value)	-<br>Note, that in manuscript it is called: enablement value / value to other interventions

Original (Panel 1) CATEGORY	Report CATEGORY	Original (Panel 1) DEFINITION	Report definition	Change
**B5. AMR prevention value**	**B5. AMR prevention value**	Impact on the rate of development and transmission of resistant bacterial infections, and associated morbidity and mortality	Impact on the rate of development and transmission of resistant bacterial infections, and associated morbidity and mortality	-
**B6. Mental health impact**	**B6. Mental health impact**	Impact on mental health and well-being of the population through avoiding non-pharmaceutical interventions impacting mental health (e.g., lockdowns, school closures)	Impact on mental health and well-being of the population directly through vaccination and indirectly through avoiding non-pharmaceutical interventions that can impact mental health (e.g., lockdowns, school closures)	Definition changed to include direct effect
**B7. Health impact of congestion externality**	**B7. Health system impact**	Impact on morbidity and mortality in the population through avoiding or mitigating overload of public health service facilities and resulting delays in diagnostic and care services	Impact of vaccination on morbidity and mortality in the population through avoiding or mitigating overload of public health service facilities and resulting delays in diagnostic and care services	Category name changed, definition slightly changed
**B8. Health equity value**	**B8. Health equity value**	The absence of disparities in health	Vaccination's impact on disparities in health	Definition changed to emphasize vaccination
**C. Effect on public finances**	**C. Effect on public finances**	The costs of vaccination and its cost offsets to public finances	The costs of vaccination and its cost offsets to public finances	-
**C1. Cost offsets to health care system**<br>**C1.1 Avoided care cost of infected patients**<br>**C1.2 Avoided care cost related to broad health effects**	**C1. Cost offsets to health care system**<br>**C1.1 Avoided care cost of infected patients**<br>**C1.2 Avoided care cost related to broad health effects**	As with many interventions, vaccines come at a cost to the health care system and also generate cost offsets by avoiding downstream health care consumption.<br>• C1.1 The value of avoiding the excess costs of treatment of more severe cases<br>• C1.2 The value of avoiding costs related to broad health effects including mental health care costs and extra care costs related to delayed diagnosis and care due to congestion externality	As with many interventions, vaccines come at a cost to the health care system and also generate cost offsets by avoiding downstream health care consumption.<br>• C1.1 The value of avoiding the excess costs of treatment of more severe cases<br>• C1.2 The value of avoiding costs related to broad health effects including mental health care costs and extra care costs related to delayed diagnosis and care due to health system impact	-
**C2. Financial sustainability and programmatic synergies**	**C2. Financial sustainability and programmatic synergies**	Improved financial sustainability of health care programs as a result of synergies with vaccination programs and/or stimulation of private demand. (Deogaonkar 2012)	Improved financial sustainability of health care programs as a result of synergies with vaccination programs and/or stimulation of private demand. (Definition based on Deogaonkar, 2012)	-
**C3. Public sector budget impact**	**C3. Public sector budget impact**	Impact on government revenues (e.g., taxes and social security contributions) and expenditures (e.g., transfers including sick benefit) related to	Impact on government revenues (e.g., taxes and social security contributions) and expenditures (e.g., transfers including sick benefit) related to	-

Original (Panel 1) CATEGORY	Report CATEGORY	Original (Panel 1) DEFINITION	Report definition	Change
		the productivity impact (D1) and macroeconomic effects (D5) corresponding to the effect of vaccination on morbidity and mortality, and on the level of non-pharmaceutical interventions.	the productivity impact (D1) and macroeconomic effects (D5) corresponding to the effect of vaccination on morbidity and mortality, and on the level of non-pharmaceutical interventions.	
**D. Societal and economic effects**	**D. Societal and economic effects**	Economic impact of vaccines outside of the public sector	Economic impact of vaccines outside of the public sector	-
**D1. Productivity impact**<br>**D1.1 Impact on patient productivity**<br>**D1.2 Impact on carer productivity**<br>**D1.3 Impact on productivity of other individuals**	**D1. Productivity impact**<br>**D1.1 Impact on patient productivity**<br>**D1.2 Impact on carer productivity**<br>**D1.3 Impact on productivity of other individuals**	• D1.1 Impact on lost days of work and on the level of productivity at work, both for getting vaccinated and for disease or mortality avoided<br>• D1.2 Impact on caregivers' time spent and level of productivity at work due to caring for a patient or taking them to be vaccinated<br>• D1.3 Impact on lost days of work and reduced productivity through contribution to avoiding non-pharmaceutical interventions (e.g., lockdowns preventing work or impacting work efficiency, school closures decreasing parents' working hours and productivity)	• D1.1 Impact on lost days of work and on the level of productivity at work, both for getting vaccinated and for disease or mortality avoided<br>• D1.2 Impact on caregivers' time spent and level of productivity at work due to caring for a patient or taking them to be vaccinated<br>• D1.3 Impact on lost days of work and reduced productivity through contribution to avoiding non-pharmaceutical interventions (e.g., lockdowns preventing work or impacting work efficiency, school closures decreasing parents' working hours and productivity)	-
**D2. Impact on costs of non-pharmaceutical interventions**	**D2. Impact on costs of non-pharmaceutical interventions**	Reduction or elimination of the need for, and hence the costs of non-pharmaceutical interventions designed to contain disease outbreaks, epidemics, or pandemics (e.g., lockdowns, use of face masks)	Reduction or elimination of the need for, and hence the costs of non-pharmaceutical interventions designed to contain disease outbreaks, epidemics, or pandemics (e.g., lockdowns, use of face masks)	-
**D3. Impact on foregone education**<br>**D3.1 Impact on foregone education of patient**<br>**D3.2 Impact on foregone education of other individuals**	**D3. Impact on foregone education**<br>**D3.1 Impact on foregone education of patient**<br>**D3.2 Impact on foregone education of other individuals**	Contribution to the avoidance of lost school days due to illness or school closures related to disease containment measures	Contribution to the avoidance of lost school days directly due to illness or indirectly through school closures (related to disease containment measures)	Definition changed to include direct / indirect effects

Original (Panel 1) CATEGORY	Report CATEGORY	Original (Panel 1) DEFINITION	Report definition	Change
**D4. Changes in household behaviour**	**D4. Changes in individual and household behaviour**	Economic improvements due to changes in household choices such as fertility and consumption/savings as a result of vaccination	Consequences of changes in household choices as a result of vaccination, including areas of fertility, consumption/savings, attitude towards risk of infection and infection control measures, willingness to vaccinate against other diseases	Both category name and definition changed to reflect individual behaviour as well
**D5. Macroeconomic effects**	**D5. Macroeconomic effects**	Reduction or elimination of the macroeconomic impact of lost productivity and non-pharmaceutical interventions designed to contain disease outbreaks, epidemics, or pandemics (Note, macroeconomic effects are affected by D4.)	Reduction or elimination of the macroeconomic impact of lost productivity and non-pharmaceutical interventions designed to contain disease outbreaks, epidemics, or pandemics (Note, macroeconomic effects are affected by D4.)	-
**D6. Income equity value**	**D6. Income equity value**	Reduction or elimination of the impact of the disease and of non-pharmaceutical interventions designed to contain it on the income distribution	Reduction or elimination of the impact of the disease and of non-pharmaceutical interventions designed to contain it on the income distribution	-
**D7. Scientific spill-over effects**	**D7. Scientific spill-over effects**	The impact of research and development on our collective knowledge, arising when innovators cannot entirely appropriate the benefit of scientific advances	The impact of research and development on our collective knowledge, arising when innovators (vaccine developers) cannot entirely appropriate the benefit of scientific advances	Minor definition change
**D8. Environmental effects**	**D8. Environmental effects**	The effect the additional waste generated by vaccination exerts on the environment, and the effect on air and water pollution, and waste generation through impact on productivity and the level of non-pharmaceutical interventions, including widespread use of disposable items	The effect the additional waste generated by vaccination exerts on the environment, and the effect on air and water pollution, and waste generation through impact on productivity and the level of non-pharmaceutical interventions, including widespread use of disposable items	-
**E. Uncertainty value**	**E. Uncertainty value**	The values generated by different concepts revolving around uncertainty.	The values generated by different concepts revolving around uncertainty.	-
**E1. Insurance value**	**E1. Insurance value**	The value to vaccinated individuals of being protected from the physical and financial burden of an illness. It has two components: vaccination reduces the ‘physical risk’ of getting sick and vaccination expands the possibilities for insuring against illness (‘financial risk protection’) - Lakdawalla (2018)	The value to vaccinated individuals of being protected from the physical and financial burden of an illness. It has two components: vaccination reduces the ‘physical risk’ of getting sick and vaccination expands the possibilities for insuring against illness (‘financial risk protection’) - Definition based on Lakdawalla et al 2018	-
**E2. Real option value**	**E2. Real option value**	Opportunities created for the patient to benefit from future advances in medicine by extending their life	Opportunities created for the patient to benefit from future advances in medicine by extending their life Definition based on Lakdawalla et al 2018	-

Original (Panel 1) CATEGORY	Report CATEGORY	Original (Panel 1) DEFINITION	Report definition	Change
**E3. Psychological benefits related to reduced uncertainty**<br>**E3.1 Value of hope**<br>**E3.2 Value of knowing**<br>**E3.3 Fear of diseases / contagion**	**E3. Psychological effects related to uncertainty**<br>**E3.1 Value of hope**<br>**E3.2 Value of knowing**<br>**E3.3 Fear of diseases / contagion**<br>**E3.4 Vaccine anxiety**	• E3.1 Impact of reducing probability of illness on patients' utility who may value a treatment/intervention with high variability in outcomes (e.g., a severely ill patient undertaking a risky procedure for a low probability chance of a cure) or may prefer a treatment/intervention with less variability around expected outcomes (e.g., a COVID-19 vaccine lowering the chance of hospitalization)<br>• E3.2 Impact on patients' utility who may attach value from the knowledge that a certain diagnosis will predict treatment effectiveness.<br>• E3.3 The value of reducing the anxiety of a (future) spread of a disease	• E3.1 Impact of reducing probability of illness on patients' utility who may value an intervention with high variability in outcomes (e.g., a severely ill patient undertaking a risky procedure for a low probability chance of a cure) or may prefer a treatment/intervention with less variability around expected outcomes (e.g., a COVID-19 vaccine lowering the chance of hospitalization) Definition based on Lakdawalla et al 2018<br>• E3.2 Impact on patients' utility who may attach value to the knowledge that a vaccine will impact the probability of illness and severe outcomes Definition based on Lakdawalla et al 2018<br>• E3.3 The value of reducing the anxiety of a (future) spread of a disease Definition based on Lakdawalla et al 2018<br>• E3.4 The negative psychological effect related to the actual and/or perceived risk of vaccine adverse effects	E3.4 Vaccine anxiety added, E3.2 definition changed for clarity

AMR, antimicrobial resistance; COVID-19, coronavirus disease 2019; DALY, disability-adjusted life years; GDP, gross domestic product; QALY, quality-adjusted life years; QoL, quality of life; R&D, research and development
