# Supplementary material for: The Societal Value of Vaccines: Expert-Based Conceptual Framework and Methods Using COVID-19 Vaccines as a Case Study
Source: Vaccines (Basel). 2023 Jan 20;11(2):234. doi: 10.3390/vaccines11020234 (PMC9961127; doi:10.3390/vaccines11020234)
Supplement: Supplementary file 1 [file vaccines-11-00234-s001.zip › Supplementary material_S3.pdf]

Supplementary Material S3: 'Quality of Evidence' linking the value elements to COVID-19 and/or vaccination and 'Ability to include' the value elements in the framework.

The evaluation was based on a targeted literature review and subjective judgment, as opposed to systematic literature reviews, and strictly defined standardised criteria. It should only be regarded as an indicative starting point for expert discussion. Similar evaluations were presented for certain value elements in Jit *et al*. [2], and the OHE Consulting Report 2021 [3], where expert opinion was inferred using the Delphi method.

As shown in the below Table, Ability reflected the availability of methods for inclusion in quantitative vaccine assessments – even value elements without monetisable value can be reflected in qualitative assessments. The evidence identified was in some cases empirical, whereas in other cases only based on model estimates. While all evidence are subject to uncertainties arising from potential measurement errors, estimates from economic models are based on underlying model assumptions, which is an additional source of uncertainty. Further, literature is quickly evolving, hence the low level of evidence for certain value items may improve over time.

Table S2: Evidence Appraisal

**Evidence:** is there good quality evidence about causal pathways to broader impact?	• Likelihood of causal relationship based on available identified evidence and/or expert judgment<br>• Quality of identified empirical evidence<br>• Quantitative size of the effect	**High (1)**<br>Strong causal relationship, high quality of evidence for COVID impact AND vaccine effect	**Moderate (2)**<br>Moderate causal relationship and moderate quality of evidence for COVID OR vaccine effect	**Low (3)**<br>Very limited evidence and unclear direction, small or no effects identified
**Ability:** is it feasible to estimate the $/QALY impact?	• Availability of monetary value of the element<br>• Timeframe over which the effect is expected to manifest (short-term usually has lower uncertainty)	**High (1)**<br>Can be monetised (method exists)	**Moderate (2)**<br>Can be monetised (method exists) but with strong assumptions and/or long term only	**Low (3)**<br>No monetary value or very high uncertainty

COVID-19, coronavirus disease 2019; QALY, quality-adjusted life years

REFERENCES

1. Centre for Reviews and Dissemination (CRD), Systematic reviews: CRD's guidance for undertaking reviews in health care. 2009.
2. Jit M, Hutubessy R. Methodological Challenges to Economic Evaluations of Vaccines: Is a Common Approach Still Possible? Appl Health Econ Health Policy. 2016;14(3):245-52.
3. Brassel S, Neri, M., and Steuten, L.,. Realising the Value of Vaccines in the UK: Ready for Prime Time? OHE Consulting Report, London: Office of Health Economics 2021 [Available from: https://www.ohe.org/publications/realising-broader-value-vaccines-uk-ready-prime-time .
