# Supplementary material for: The Societal Value of Vaccines: Expert-Based Conceptual Framework and Methods Using COVID-19 Vaccines as a Case Study
Source: Vaccines (Basel). 2023 Jan 20;11(2):234. doi: 10.3390/vaccines11020234 (PMC9961127; doi:10.3390/vaccines11020234)
Supplement: Supplementary file 1 [file vaccines-11-00234-s001.zip › Supplementary material_S4.pdf]

Supplementary Material S4: Summary of the evidence review and gap analysis

Figure S1 Evidence Review and Gap Analysis (1/3)

BRAVE	VALUE CATEGORY	DESCRIPTION	BRAVE (BELL 2022)	EVIDENCE	ABILITY
A	A. Narrow Health Effects	**Impact of vaccines on the health of vaccinated individuals**			
	A1. Impact on length of life and QoL of patients	Impact of vaccines on life expectancy or life-years saved, and on patients' physical, mental, emotional, and social functioning	√	1	1
B	B. Broad Health Effects	**Impact of vaccines on the health of the unvaccinated population**			
	B1. Impact on QoL<br>B1.1 Impact on QoL of carers<br>B1.2 Impact on QoL of other individuals	Impact of vaccines on caregivers' and other individuals' physical, mental, emotional, and social functioning	√<br>N	1<br>3	1<br>2
	B2. Transmission value	Impact of vaccination on disease transmission patterns and associated morbidity and mortality	√	1	1
	B3. Burden of disease	Impact on overall burden of disease to society, in terms of prevalence and severity, estimated through the total amount of associated morbidity and mortality (Note, this includes A1., B1., and B2.)	√	1	1
	B4. Value to other interventions / enablement value	Impact of vaccination on the cost effectiveness of other non-vaccine interventions	√	3	2
	B5. AMR prevention value	Impact on the rate of development and transmission of resistant bacterial infections, and associated morbidity and mortality. Preventing infectious disease through vaccination reduces antibiotic use and therefore reduces antimicrobial resistance.	√	3	3
	B6. Mental health impact	Impact of vaccination on mental health and well-being of the population through avoiding non-pharmaceutical interventions impacting mental health (e.g., lockdowns, school closures). This may in turn also reduce prevalence of other disorders (anxiety, substance abuse, stress disorders) that are exacerbated by a pandemic.	Not explicitly	1	1
	B7. Health system impact	Impact on morbidity and mortality in the population through avoiding or mitigating overload of public health service facilities and resulting delays in diagnostic and care services	N	1	2
	B8. Health equity value	Vaccination can lead to more equal distribution of health outcomes.	N	2	3

High (1) | Moderate (2) | Low (3)

AMR, antimicrobial resistance; QoL, quality of life

Figure S2 Evidence Review and Gap analysis (2/3)

BRAVE	VALUE CATEGORY	DESCRIPTION	BRAVE (BELL 2022)	EVIDENCE	ABILITY
C	**C. Effect on public finances**	**The costs of vaccination and its cost offsets to public finances**			
	**C1. Cost offsets to health care system**<br>**C1.1 Avoided care cost of infected patients**<br>**C1.2 Avoided care cost related to broad health effects**	C1. Impact on medical costs borne by the health system from potential reductions in the number of general practitioner and specialist consultations, treatment, screening interventions, and hospitalizations<br>C1.1 The value of avoiding the excess costs of treatment of more severe cases<br>C1.2 The value of avoiding costs related to broad health effects including mental health care costs and extra care costs related to delayed diagnosis and care due to congestion externality	√<br>N	1<br>2	1<br>2
	**C2. Financial sustainability and programmatic synergies**	Improved financial sustainability of health care programs as a result of synergies with vaccination programs and/or stimulation of private demand.	N	3	1
	**C3. Public sector budget impact**	Impact on government revenues (e.g., taxes and social security contributions) and expenditures (e.g., transfers including sick benefit) related to the productivity impact (D1) and macroeconomic effects (D5) corresponding to the effect of vaccination on morbidity and mortality, and on the level of non-pharmaceutical interventions.	√	1	2
D	**D. Societal and economic effects**	**Economic impact of vaccines outside of the public sector**			
	**D1. Productivity impact**<br>**D1.1 Impact on patient productivity**<br>**D1.2 Impact on carer productivity**<br>**D1.3 Impact on productivity of other individuals**	D1.1 Impact on lost days of work and on the level of productivity at work, both for getting vaccinated and for disease or mortality avoided<br>D1.2 Impact on caregivers' time spent and level of productivity at work due to caring for a patient or taking them to be vaccinated<br>D1.3 Impact on lost days of work and reduced productivity through contribution to avoiding non-pharmaceutical interventions (e.g., lockdowns preventing work or impacting work efficiency, school closures decreasing parents' working hours and productivity)	√<br>√<br>N	1<br>1<br>2	1<br>1<br>2
	**D2. Impact on costs of non-pharmaceutical interventions**	Reduction or elimination of the need for, and hence the costs of non-pharmaceutical interventions designed to contain disease outbreaks, epidemics, or pandemics (e.g., lockdowns, use of face masks)	N	3	2
	**D3. Impact on foregone education**<br>**D3.1 Impact on foregone education of patient**<br>**D3.2 Impact on foregone education of other individuals**	Contribution to the avoidance of lost school days due to illness or school closures related to disease containment measures	N<br>N	1<br>2	3<br>3

High (1) | Moderate (2) | Low (3)

Figure S3 Evidence Review and Gap Analysis (3/3)

BRAVE	VALUE CATEGORY	DESCRIPTION	BRAVE (BELL 2022)	EVIDENCE	ABILITY
D	**D4. Changes in household behaviour**	Economic improvements due to changes in household choices such as fertility and consumption/savings as a result of vaccination	N	3	3
D	**D5. Macroeconomic effects**	Reduction or elimination of the macroeconomic impact of lost productivity and non-pharmaceutical interventions designed to contain disease outbreaks, epidemics, or pandemics (Note, macroeconomic effects are affected by D4.)	√	1	2
D	**D6. Income equity value**	Reduction or elimination of the impact of the disease and of non-pharmaceutical interventions designed to contain it on the income distribution	√	2	3
D	**D7. Scientific spill-over effects**	The impact of research and development on our collective knowledge, arising when innovators cannot entirely appropriate the benefit of scientific advances	N	3	3
D	**D8. Environmental effects**	The effect the additional waste generated by vaccination exerts on the environment, and the effect on air and water pollution, and waste generation through impact on productivity and the level of non-pharmaceutical interventions, including widespread use of disposable items	N	2	2
E	**E. Uncertainty value**	The values generated by different concepts revolving around uncertainty.			
E	**E1. Insurance value**	The value to vaccinated individuals of being protected from the physical and financial burden of an illness. It has two components: vaccination reduces the 'physical risk' of getting sick and vaccination expands the possibilities for insuring against illness ('financial risk protection')	N	3	2
E	**E2. Real option value**	Vaccination's impact on opportunities created for the patient to benefit from future advances in medicine by extending their life	N	3	3
E<br>NEW	**E3. Psychological benefits related to reduced uncertainty**<br>**E3.1 Value of hope**<br>**E3.2 Value of knowing**<br>**E3.3 Fear of diseases / contagion**	E3.1 Impact of reducing probability of illness on patients' utility who may value a treatment/intervention with high variability in outcomes (e.g., a severely ill patient undertaking a risky procedure for a low probability chance of a cure) or may prefer a treatment/intervention with less variability around expected outcomes (e.g., a COVID-19 vaccine lowering the chance of hospitalization)<br>E3.2 Impact on patients' utility who may attach value to the knowledge that a certain diagnosis will predict treatment effectiveness.<br>E3.3 The value of reducing the anxiety of a (future) spread of a disease	N<br>N<br>Not explicitly	3<br>3<br>3	3<br>3<br>3

High (1) | Moderate (2) | Low (3)

COVID-19, coronavirus disease 2019
