# Supplementary material for: The Societal Value of Vaccines: Expert-Based Conceptual Framework and Methods Using COVID-19 Vaccines as a Case Study
Source: Vaccines (Basel). 2023 Jan 20;11(2):234. doi: 10.3390/vaccines11020234 (PMC9961127; doi:10.3390/vaccines11020234)
Supplement: Supplementary file 1 [file vaccines-11-00234-s001.zip › Supplementary material_S5.pdf]

## Supplementary Material S5: Identified Quantification Methods for Broader Value Elements and Expert Elicitation on their Appropriateness.

### Table S3 Quantification methods and Expert Elicitation

| Broader Value Element and Quantification Method - Literature Findings | Round 1 Polling (N=8) | | Experts' Views during the Panel 2 Discussions |
|---|---|---|---|
| | % High rank (4 or 5) | % Low rank (1 or 2) | |
| ***B: Broader Health Effects; C: Effects on Public Finances*** | | | |
| **B5. AMR – extensions to CEA or CBA** | 50% | 25% | - Experts stated that although antimicrobial resistance (AMR) was not ranked high, it might emerge in the future debates as new evidence will be available. |
| Although the pandemic has influenced antibiotic use, both directly through antibiotics rx or indirectly by influencing access to antibiotics of other patients [4], no method was identified that has been applied to the quantification of the relationship between the pandemic and AMR. | | | |
| **B6. & C1.2. Mental health impact – Approach based on number of additional depression cases** | 38% | 50% | |
| The mental health impact of the pandemic and the related social restrictions were widely documented, two approaches were identified, wherein, monetary value was attached to the mental impact by multiplying excess case numbers by average cost of treatment per person [5]. | | | |
| **B6. & C1.2. Mental health impact – Approach based on impact of vaccination on months spent in depression** | 38% | 50% | |
| In the second approach, authors multiplied the similarly calculated severity-specific excess depression case numbers by QoL, and healthcare cost impact associated with each depression severity category [6]. | | | |
| **B7. & C1.2 Health system impact – Opportunity cost** | 50% | 50% | |

| Broader Value Element and Quantification Method - Literature Findings | Round 1 Polling (N=8) | | Experts' Views during the Panel 2 Discussions |
|---|---|---|---|
| | % High rank (4 or 5) | % Low rank (1 or 2) | |
| The disruption in service provision has been documented via a variety of outcome measures including backlogs, waiting times, the share of incomplete patient pathways etc. [3, 7-9] or on the demand side such as an estimate on the number of people that need care but have not yet come forward to receive care due to the pandemic [7]. Monetising the impact of this congestion externality used the foregone net monetary benefit associated with the treatments as the opportunity cost associated with inpatient bed-days [10]. | | | - The methodology for assessing the monetary value of the health system impact by evaluating healthcare resources by their opportunity cost as opposed to their accounting costs was considered appropriate and could be extended further to cover foregone screenings. This direct approach concentrated on the effect of the congestion externality and covered both mortality and QoL implications.<br>- Using excess deaths as a measure for the indirect mortality impact of COVID-19—including but not limited to the effect of health system congestion—is controversial. It is a simple but powerful method for capturing indirect mortality impact in a comprehensive way, and during periods where no other major change occurred that could substantially impact mortality, it is reasonable to assign all change in mortality to the pandemic. Some of the major confounding factors involved in these analyses, such as the reaping effect and mortality displacement may completely or partially be adjusted for by age standardisation and other statistical techniques.<br>- In forward looking analyses, a multiplier capturing the relationship between ICU case numbers and excess inpatient deaths could be used for predicting excess deaths based on case numbers.<br>- Excess deaths should only be assumed during time periods when demand for health services exceeds capacity. |
| While certain elements of the public finance impact of the pandemic have been estimated for both the US [11] and UK [12], no study was identified calculating the full public finance impact or the return on investment of the COVID-19 vaccination. | | | |
| **C3. Public finance impact – ROI** | 63% | 13% | |
| **C3. Public finance impact – fBCR** | 50% | 13% | |
| ***D: Societal and Economic Effects*** | | | |
| **D1. Productivity loss - Human capital** | 50% | 13% | - Human capital approach captures lost income due to mortality and morbidity associated with a disease at an individual level, but at an economic level, the structure of the labour market needs to be accounted for. |
| | | | |
| **D1. Productivity loss - Friction cost** | 50% | 25% | |

| Broader Value Element and Quantification Method - Literature Findings | Round 1 Polling (N=8) | | Experts' Views during the Panel 2 Discussions |
|---|---|---|---|
| | % High rank (4 or 5) | % Low rank (1 or 2) | |
| **D2. Impact on the cost of NPIs – Approach based on relationship between vaccination and NPI levels**<br>No identified work estimated the impact of vaccination on the direct cost of NPIs. A possible approach to quantifying this impact is to assess the change in resource use (e.g., in the number of face masks used) as a result of vaccination and multiply that by the unit cost. | 38% | 25% | - The friction cost method usually gives a smaller value estimate and is more appropriate in most situations.<br>- The loss of firm-specific human capital associated with losing job and getting re-employed is potentially bigger than the loss associated with time spent off work and hence needs to be accounted for, alongside cross-country differences in labour market conditions.<br>- Aspects of productivity are not generally considered in assessments include the reorganisation of production resulting from the pandemic, and the fact that losing one's job and getting re-employed is associated with a substantial loss of firm-specific human capital. |
| **D3. Education loss – Approach based on impact on test scores**<br>The OECD approach for assessing the impact of lost education on individual income and GDP [13], based on the impact of school closures on test scores (proxy for lost cognitive ability), and its relation to decrease in lifetime earnings. | 63% | 38% | - OECD approach was considered straightforward and worthy of conduction.<br>- Distributional consequences were also considered important to capture, and impacts need to be assessed across different education levels too.<br>-In assessments including macroeconomic impact, the estimated GDP impact associated with school closures needs to be reconciled with GDP changes arising from other factors to avoid double counting.<br>- Besides school closures, online education periods may also be accounted for by proxying the reduction in efficacy of online schooling compared to offline schooling based on assessments of work from home capabilities based on telecommunication infrastructure. |
| **D3. Education loss – Microsimulation**<br>The Penn Wharton Budget Model, an individual-level stochastic simulation model of labour productivity, in which the effect of lost education is incorporated through grade-specific achievement score gains that are converted into an estimate of loss in effective years of schooling [14]. | 75% | 13% | |
| **D5. Impact on GDP – Approach relies on external estimates for GDP** | 38% | 13% | - Avoiding NPIs and concentrating on GDP (or gross value added) as the only macroeconomic outcome measure is an appropriate |

<table>
<tr><th rowspan="2">Broader Value Element and Quantification Method - Literature Findings</th><th colspan="2">Round 1 Polling (N=8)</th><th rowspan="2">Experts' Views during the Panel 2 Discussions</th></tr>
<tr><th>% High rank (4 or 5)</th><th>% Low rank (1 or 2)</th></tr>
<tr><td>Literature assessing the various aspects of the COVID-19 pandemic's macroeconomic impact was widely documented, mainly concentrating on GDP and employment as outcome measures, with 3 approaches. Certain studies relied on publicly available GDP estimates from other entities, such as the Congressional Budget Office [11] or Goldman Sachs [6].</td><td></td><td></td><td rowspan="5">approach for synthesising macroeconomic impact without double-counting. It can be done either by directly estimating a multiplier of case numbers on GDP directly or by taking a two-step approach.<br>- The two-step approach involves estimating the multiplier between inpatient case numbers and NPI levels to capture the policy responses to decreasing case numbers as the policy is expected to be more responsive to impact on hospital capacities than on mortality, and then estimating another multiplier between NPI levels and GVA.<br>- When assessing impact on production and value added, it is important to cover non-market production too.<br>- Besides overall GDP, that is not sensitive to distributional outcomes, measures of macroeconomic performance should also consider impact on health equity and income inequalities through social welfare measures. While macroeconomic impact is important under pandemic settings, building de novo macroeconomic or combined epidemiological and macroeconomic models do not seem appropriate for HTA purposes, due to their complexity and inherent uncertainties.</td></tr>
<tr><td>D5. Impact on GDP – Simple estimate using time series data</td><td rowspan="2">38%</td><td rowspan="2">25%</td></tr>
<tr><td>Other works performed simple time-series analyses comparing pre- and post-pandemic GDP levels [15].</td></tr>
<tr><td>D5. Impact on GDP – Macroeconomic modelling</td><td rowspan="2">63%</td><td rowspan="2">0%</td></tr>
<tr><td>Based on the macroeconomic models, such as computable general equilibrium models or combined epidemiological and macroeconomic models [16-18].</td></tr>
<tr><td>D8. Environmental effects</td><td rowspan="2">NR</td><td rowspan="2">NR</td><td rowspan="2"></td></tr>
<tr><td>Various aspects of the environmental impact of the pandemic have been documented in the literature [19]. Positive impacts, such as air quality improvement, reduction in water and noise pollution, or getting closer to sustainable development goals are arising from the lower industrial activity and less traffic related to the disruption in economic activities. Negative impacts include biomedical waste generation, increased municipal waste generation, and a</td></tr>
</table>

| Broader Value Element and Quantification Method - Literature Findings | Round 1 Polling (N=8) | | Experts' Views during the Panel 2 Discussions |
|---|---|---|---|
| | % High rank (4 or 5) | % Low rank (1 or 2) | |
| parallel decrease in waste recycling in certain countries. No identified study estimated the monetary impact of these elements. | | | |
| ***E: Uncertainty Value*** | | | |
| **E3. Psychological effects**<br>Increasing rates of the COVID-19 vaccination rates have shown psychological benefits, measured by lower levels of anxiety, worry, displeasure, and depression in the US. However, no identified study had attached monetary value to the positive or negative psychological value of vaccination. | NR | NR | - Psychological effects of vaccinations encompassing both the positive and negative elements, are important to account for in epidemiology models as they have an important impact on uptake and hence on infection numbers.<br>- Vaccine mandates impose cost on people through vaccine anxiety that should be included at least qualitatively but potentially even quantitatively. Including their direct QALY impact would increase complexity, likely without substantially changing conclusions.<br>- In some US states payments were offered for taking the vaccine, but these policies did not achieve big impact, suggesting the anxiety to potentially be substantive - which can be a topic for further research.<br>- Methods for quantifying insurance value and AMR prevention value are under development but no established best practice is available. For measuring vaccine anxiety, both stated preference and revealed preference approaches may be feasible. |

AMR, antimicrobial resistance; CBA, cost benefit analysis; CEA, cost effectiveness analysis; COVID-19, coronavirus disease 2019; DALY, disability-adjusted life years; fBCR, fiscal benefit to cost ratio; GDP, gross domestic product; GVA, gross value added; QALY, quality-adjusted life years; QoL, quality of life; NPI, non-pharmaceutical intervention; NR, not reported; OECD, Organisation for Economic Co-operation and Development; ROI, return on investment; UK, United Kingdom; US, United States

## REFERENCES


1. Centre for Reviews and Dissemination (CRD), Systematic reviews: CRD's guidance for undertaking reviews in health care. 2009.
2. Jit M, Hutubessy R. Methodological Challenges to Economic Evaluations of Vaccines: Is a Common Approach Still Possible? Appl Health Econ Health Policy. 2016;14(3):245-52.
3. Brassel S, Neri, M., and Steuten, L.,. Realising the Value of Vaccines in the UK: Ready for Prime Time? OHE Consulting Report, London: Office of Health Economics 2021 [Available from: https://www.ohe.org/publications/realising-broader-value-vaccines-uk-ready-prime-time .
4. Sevilla JP, Bloom DE, Cadarette D, Jit M, Lipsitch M. Toward economic evaluation of the value of vaccines and other health technologies in addressing AMR. Proceedings of the National Academy of Sciences. 2018;115(51):12911-9.
5. Cutler DM, Summers LH. The COVID-19 Pandemic and the $16 Trillion Virus. JAMA. 2020;324(15):1495-6.
6. Kirson N, Swallow E, Lu J, et al. The societal economic value of COVID-19 vaccines in the United States. J Med Econ. 2022;25(1):119-28.
7. Lane Clark & Peacock (LCP). Hidden health needs 'the elephant in the NHS waiting room' as waiting list number could rise to over 15 million in 2023 2021 [Available from: https://www.lcp.uk.com/media-centre/2021/12/hidden-health-needs-the-elephant-in-the-nhs-waiting-room-as-waiting-list-number-could-rise-to-over-15-million-in-2023/.
8. Mayo M, Potugari B, Bzeih R, et al. Cancer Screening During the COVID-19 Pandemic: A Systematic Review and Meta-analysis. Mayo Clin Proc Innov Qual Outcomes. 2021;5(6):1109-17.
9. The Health Foundation. Health and social care funding to 2024/25. Slide deck of key findings 2021 [Available from: https://www.health.org.uk/publications/reports/health-and-social-care-funding-to-2024-25.
10. Brassel S, Neri M, Schirrmacher H, Steuten L. The Value of Vaccines in Maintaining Health System Capacity in England. Office of Health Economics; 2021.
11. Congressional Budget Office. Budgetary Effects of the 2020 Coronavirus Pandemic 2020 [Available from: https://www.cbo.gov/publication/56388.
12. Heald D, Hodges R. The accounting, budgeting and fiscal impact of COVID-19 on the United Kingdom. Journal of Public Budgeting, Accounting & Financial Management. 2020.
13. Hanushek EA, Woessmann L. The economic impacts of learning losses. 2020.
14. Penn Wharton, University of Pennsylvania. COVID-19 Learning Loss: Long-run Macroeconomic Effects Update 2021 [Available from: https://budgetmodel.wharton.upenn.edu/issues/2021/10/27/covid-19-learning-loss-long-run-macro-effects.
15. Sandmann FG, Davies NG, Vassall A, et al. The potential health and economic value of SARS-CoV-2 vaccination alongside physical distancing in the UK: a transmission model-based future scenario analysis and economic evaluation. Lancet Infect Dis. 2021;21(7):962-74.
16. Arnon A, Ricco J, Smetters K. Epidemiological and economic effects of lockdown. Brookings Papers on Economic Activity. 2020;2020(3):61-108.
17. Choi Y, Kim H-j, Lee Y. Economic consequences of the COVID-19 pandemic: will it be a barrier to achieving sustainability? Sustainability. 2022;14(3):1629.
18. Chudik A, Mohaddes K, Pesaran MH, Raissi M, Rebucci A. A counterfactual economic analysis of Covid-19 using a threshold augmented multi-country model. J Int Money Finance. 2021;119:102477.
19. Singh V, Mishra V. Environmental impacts of coronavirus disease 2019 (COVID-19). Bioresour Technol Rep. 2021;15:100744.
